# Vibration Analysis of Shape Memory Alloy Enhanced Multi-Layered Composite Beams with Asymmetric Material Behavior

**DOI:** 10.3390/ma18051181

**Published:** 2025-03-06

**Authors:** Kosar Samadi-Aghdam, Pouya Fahimi, Hamid Shahsavari, Davood Rahmatabadi, Mostafa Baghani

**Affiliations:** 1 School of Mechanical Engineering, College of Engineering, University of Tehran, Tehran 14155-6619, Iran; kosar.samadi@ut.ac.ir (K.S.-A.); d.rahmatabadi@ut.ac.ir (D.R.); 2 University of Oklahoma Health Sciences Center, Oklahoma City, OK 73104, USA; hamid.shahsavari@kit.edu

**Keywords:** shape memory alloys, composite beam, tension–compression asymmetry, hysteresis, energy-dissipating

## Abstract

This study develops a finite element solution to analyze the vibration response of multi-layer shape memory alloy (SMA) composite beams. Using Euler–Bernoulli beam motion equations with tension–compression asymmetry, based on Poorasadion’s model, the Newmark method and Newton–Raphson technique are employed. Validating the model against ABAQUS/Standard results for a homogeneous SMA beam shows good agreement. This research explores the dynamic characteristics of bi-layer and tri-layer SMA beams, presenting deflection–time, stress–strain, and velocity–deflection profiles. SMAs’ hysteresis property effectively reduces early-stage vibration amplitudes, and their energy-dissipating feature during phase transformations makes them promising for controlling dynamic performance in engineering applications.

## 1. Introduction

Delving into the mechanical traits of composite materials constitutes a central research focus [1,2,3,4,5], particularly given their extensive use in engineering. It is imperative to explore various types of these materials under diverse boundary conditions. One notably popular area of investigation involves the behavior of intelligent materials within composite structures. Among different kinds of intelligent materials, shape memory alloys are renowned for two specific features involving pseudoelasticity (PE) and shape memory effect (SME). The shape memory effect alludes to the ability of SMAs that retrieve an inelastic strain by heating the material [6]. The PE effect makes the material bear large displacement without permanent deformation [7]. These significant characteristics arise from the phase transition between the austenite and martensite as a result of changes made related to temperature or stress [8]. Benefiting from these interesting features, SMAs have been vastly used in various engineering applications, such as medical devices [9,10], automobiles and trains [11], aerospace structures [12], and robotics [13]. Moreover, because of SMA’s capability in energy dissipating, these materials can control the vibration of structures [14]. Therefore, by adding SMA members to the structures influenced by dynamic loads, the amplitude of vibration, the dynamic performance, and the vibration frequency of structures can be controlled [15].

Several models have been developed to investigate SMA materials, one of which is Brinson’s model [16] which anticipates unique behaviors of SMAs in a broad range of temperatures. However, the original version of this model had two significant limitations: it cannot predict secondary effects (i.e., SMA’s behavior is different in tension and compression), and it did not consider the internal sub-loops. As a result, many types of research have been performed to improve the original form of the Brinson model (OBM) [16]. One of which belongs to Poorasadion et al. [17], where they considered the asymmetric (ASYM) behavior of SMAs in tension and compression as well as the internal sub-loops. This model is marked as PM (Poorasadion’s Model), and the asymmetric behavior of this material is marked as ASYM in this paper.

Several studies [18,19,20] have been performed to examine the vibration response of SMA beams. Feng et al. [21] examined a vibration isolator made of SMA bars both numerically and experimentally. They confirmed the influence of phase transformation induced by stress on the dynamic characteristics of the system. Hashemi et al. [22] developed a theoretical model to evaluate the vibration behavior of SMA beams. They also accounted for the asymmetric behavior of this material in tension and compression. Jafari et al. [23] analyzed the dynamic behavior of multi-span SMA beams in various types of motions, load speeds, damping effects, and load amplitudes. In this study, the effect of the hysteresis-induced damping, the natural frequency of the beam, and variations in Young’s modulus are investigated. Zbiciak et al. [24] presented a mathematical model to express the dynamic behavior of a pseudoelastic SMA beam, where they suggested a formulation for solving the initial boundary value problem. For SMA beams based on Euler–Bernoulli theory, Jose et al. [25] presented a theoretical model to analyze a vibration isolator made of SMA bars under isothermal and non-isothermal conditions. Ozbulut et al. [26] conducted experimental tests to analyze the dynamic response of SMA wires. They conduct numerical simulations by using the data from experimental tests to show the use of SMA wires in civil engineering applications. Araki et al. [27] designed a quasi-zero-stiffness vibration isolator by using SMA bars. The use of SMA bars made it easy to achieve a large stroke length and large loading capacity. The feasibility of this system has been proved through shaking table tests. Razavilar et al. [28] reported a semi-analytical solution to analyze the free and forced vibration behavior of a cantilever SMA beam.

Moreover, there have been several studies on the vibration behavior of composite beams comprising SMA layers [29,30,31,32,33]. Katsiropoulos et al. [34] examined the damping response of fiber-reinforced composites with embedded SMA wires. The results of this study showed that increasing the volume fraction of SMA wires makes a significant change in the damping behavior of composites. Alebrahim et al. [35] investigated the dynamic behavior of a composite beam underneath a moving mass. The composite beam was reinforced with SMA wires and carbon fibers. In this study, the Timoshenko beam theory and transfer matrix method were used to solve the problem. They examined the influence of different volume fractions of SMA wires, crack depth, and mass speed on the natural frequency and deflection of the composite beam. Zhang et al. [36] conducted an experimental study to demonstrate the effects of the arrangement and temperature of SMA wire inside the composite structures on their vibration response. They proposed a theoretical model on natural frequencies for laminated composites, including SMA wires, where it was in good agreement with experimental data. Balapgol et al. [37] employed the finite element method to simulate the free vibration of a cantilever composite plate, including SMA surfaces. Their work was based on the first-order shear deformation theory, in which they assumed small strain and small displacements. Damanpack et al. [38] analyzed the capability of SMA composite beams influenced by dynamic loadings. In their study, a comprehensive analysis of the effect of temperature, pre-strain, thickness, and location of SMA layers on vibration characteristics of composite beams under a variety of dynamic loads. Yongsheng et al. [39] proposed a theoretical model to demonstrate the free and forced vibration behavior of orthotropic composite plates, including SMA fibers. They employed Brinson’s constitutive model to predict SMA fiber’s characteristics. Lau et al. [40] presented an analytical model for examining the natural frequencies of a composite beam with embedded SMA wires. The results of the analytical model are verified by means of experimental measurements. They found that at a temperature lower than the martensite finish temperature, the composite beam’s natural frequencies decreased with an increase in the number of SMA wires. Srivastava et al. [41] employed the finite element method to investigate the free vibration of SMA and E-glass fiber-reinforced composites. In their study, the influence of the fiber orientation angle and fiber volume fraction on the natural frequency of composite was analyzed. Ni et al. [42] examined the vibration characteristics of composites embedded with short SMA fibers. They investigated the effect of the addition of short SMA fibers on the vibration properties of composites. Regarding the results, there was an optimal content of SMA fibers for vibration features. Gol Zardian et al. [43] performed an experimental test to investigate the free vibration characteristic of a composite beam, including SMA wires. Chen et al. [44] investigated the mathematical model of an elastic beam that was covered by SMA layers. They analyzed the capability of this material in changing the elastic modulus, recovery stress, and natural frequency of the vibrating beam.

It should be noted that some research papers [45,46,47] considered the geometric nonlinearity of the SMA beam; however, they did not account for the ASYM behavior of SMAs in tension and compression. For instance, Ashrafi et al. [48] proposed a model to analyze the nonlinear free vibration of the composite beam, including an SMA layer. They evaluated the effect of the temperature, material length scale, and initial velocity on the vibration behavior of the sandwich composite. Kim et al. [49] studied the damping characteristics of a Ni-Ti SMA beam theoretically and experimentally. In this work, the nonlinear Hilbert transform technique is utilized to analyze the damping behavior of the SMA beam.

Based on the literature, numerous studies have been conducted to analyze the vibration of SMA materials. However, most of them have some shortcomings that significantly impact the accuracy of the model. First, most of the above-mentioned papers did not take into account the ASYM material behavior in tension and compression, and others did not analyze the vibration response of multi-layered SMA composites and just proposed a solution method for homogenous SMA beams.

In this study, a finite element method solution is proposed to analyze the vibration behavior of a multi-layered SMA composite beam with consideration of the ASYM material behavior. The main superiority of this study is introducing a comprehensive and flexible model. The most important reasons for utilizing PM are considering nonlinearity in the transformation kinetics functions and the effect of the asymmetric behavior of SMA’s in tension and compression, which enhances the accuracy of the structural model. Moreover, the proposed model is applicable for analyzing the vibration of any Euler–Bernoulli beam with various layups and geometry.

It should be mentioned that the 2D beam model in ABAQUS software (Version 2023) can only simulate a homogeneous SMA beam, but for a multi-layered SMA beam with asymmetric material behavior we need a new computer program to simulate this behavior. Importantly, this paper is an extension of our previous work about multi-layered SMA beams [7]. The previous model was only capable of capturing the behavior under static conditions; however, the present work has the ability to simulate the dynamic behavior of such beams.

The rest of this paper is arranged as follows: the section “a brief review on PM” concisely introduces the main features of PM model, and the section “beam model development” presents a finite element formulation for the vibration behavior of multi-layer SMA composite beam. The model validation and numerical results are reported in the section “numerical results”, while in the section “summary and conclusion” a summary and final remarks are reported.

## 2. A Brief Review on PM

In order to omit the limitations of OBM, Poorasadion et al. [17] improved the model to take into account the ASYM behavior of SMAs by utilizing the experimental data presented in [50,51]. Additionally, the sub-loops behavior of the material is presented in their model. A brief review is submitted here; however, a detailed explanation of the model is reported by Poorasadion et al. [17].

According to the PM, the martensite volume fraction can be expressed as follows:

(1) $$\xi =\xi_{S}^{+}+\xi_{S}^{-}+\xi_{T}$$

In which, $\xi_{S}^{+}$ and $\xi_{S}^{-}$ indicate the martensite phase induced by the stress in tension and compression, respectively, and $\xi_{T}$ represents the martensite phase induced by temperature. Moreover, based on the PM, the stress–strain equation is presented as follows:

(2) $$d\sigma =Dd\varepsilon +\Omega_{S}^{+}d\xi_{S}^{+}+\Omega_{S}^{-}d\xi_{S}^{-}+\Omega_{T}d\xi_{T}+\Theta dT$$

In which $\Omega_{S}$ and $\Omega_{T}$ are the transformation coefficients induced by the stress and temperature, respectively. Moreover, D and Θ are the elastic modulus and the thermoelastic coefficient, respectively.

Based on PM, different elastic moduli are considered for fully austenite ($D_{a}$), fully detwinned martensite in tensile loading ($D_{m}^{+}$), fully detwinned martensite in compressive loading ($D_{m}^{-}$), and fully twinned martensite ($D_{T}$). Therefore, the elastic modulus can be expressed as follows:

(3) $$D(\xi )=D_{a}+\xi_{S}^{+}\left(D_{m}^{+}-D_{a}\right)+\xi_{S}^{-}\left(D_{m}^{-}-D_{a}\right)+\xi_{T}\left(D_{m}^{T}-D_{a}\right)$$

The transformation tensor induced by stress in tension and compression is in direct relationship with the maximum transformation strain and the elastic modulus. Since $\Omega_{T}$ = 0, tensor of transformation can be presented as follows:

(4) $$\Omega^{+}\left(\xi \right)=-\varepsilon_{L}^{+}D\left(\xi \right)\;\Omega^{-}\left(\xi \right)=-\varepsilon_{L}^{-}D\left(\xi \right)$$

Equations (3) and (4) are substituted into Equation (2), and by integrating the result the following is obtained:

(5) $$\begin{array}{l} \sigma -\sigma_{0}=D\left(\xi \right)\varepsilon -D\left(\xi_{0}\right)\varepsilon_{0}+\left(\Omega^{+}\left(\xi \right)\xi_{S}^{+}+\Omega^{-}\left(\xi \right)\xi_{S}^{-}\right)\\ -\left(\Omega^{+}\left(\xi_{0}\right)\xi_{S0}^{+}+\Omega^{-}\left(\xi_{0}\right)\xi_{S0}^{-}\right)+\Theta \left(T-T_{0}\right) \end{array}$$

Equation (5) represents the asymmetric material behavior of SMAs by considering non-constant material coefficients. Parameters $\Omega^{+}$, $\Omega^{-}$, and D in Equation (5) are all functions of ξ, the martensite volume fraction. Furthermore, the tangent matrix (C) is expressed it as follows:

(6) $$C=\frac{d\sigma }{d\varepsilon }=\frac{D\left(\xi \right)}{1-\left(\varepsilon -\varepsilon_{L}^{+}\xi_{S}^{+}-\varepsilon_{L}^{-}\xi_{S}^{-}\right)\left[D_{m}^{+}\frac{d\xi_{S}^{+}}{d\sigma }+D_{m}^{-}\frac{d\xi_{S}^{-}}{d\sigma }+D_{m}^{T}\frac{d\xi_{T}}{d\sigma }+D_{a}\frac{d\xi }{d\sigma }\right]}-\Omega^{+}\left(\xi \right)\frac{d\xi_{S}^{+}}{d\sigma }-\Omega^{-}\left(\xi \right)\frac{d\xi_{S}^{-}}{d\sigma }$$

In Equation (6), $\frac{d\xi_{T}}{d\sigma }$, $\frac{d\xi }{d\sigma }$, $\frac{d\xi_{S}^{+}}{d\sigma }$, and $\frac{d\xi_{S}^{-}}{d\sigma }$ are derivatives of ξ. As formerly mentioned, details of the transformation kinetics and transformation process can be found in Poorasadion et al. [17].

## 3. Beam Model Development

In this section, a formulation is proposed for the vibration of an SMA multi-layer composite beam. In the beginning, it should be mentioned that due to the nonlinear nature of this material, each layer of the SMA beam has different properties. As a result, we divided the SMA beam into a practical number of layers. Accordingly, we considered the EBT assumptions and utilized a numerical solution to achieve accurate results.

**Figure 1:**
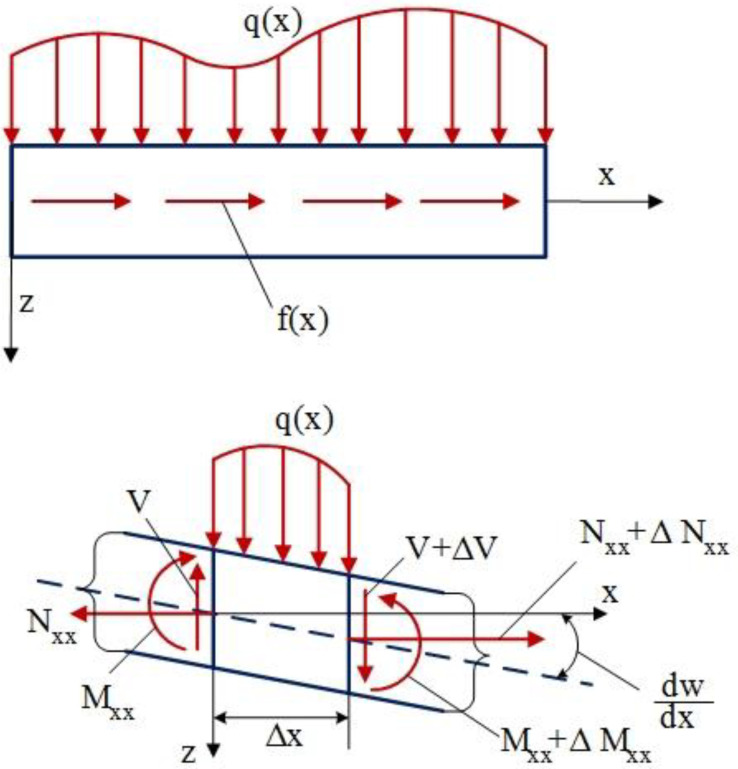
A typical beam element with forces and moments to derive motion equations.

Employing a typical beam element with all forces and moments, the equation of motion can be achieved. Considering the beam element shown in Figure 1, $q\left(x\right)$ is the external distributed transverse load and $f\left(x\right)$ denotes the external axial force. Furthermore, $N_{xx}$ and $V_{x}$ stand for the internal axial and vertical shear forces, respectively, and $M_{xx}$ indicates an internal bending moment in the beam element, the equation of motion can be expressed as follows:

(7) $$\sum F_{x}=\rho A\Delta x\frac{\partial^{2}u}{\partial t^{2}}:\;-N_{xx}+(N_{xx}+\Delta N_{xx})+f(x)\Delta x=\rho A\Delta x\frac{\partial^{2}u}{\partial t^{2}}$$

(8) $$\sum F_{z}=\rho A\Delta x\frac{\partial^{2}w}{\partial t^{2}}:\;-V+\left(V+\Delta V\right)+q(x)\Delta x=\rho A\Delta x\frac{\partial^{2}w}{\partial t^{2}}$$

(9) $$\begin{array}{l} \sum M_{y}=I\frac{\partial^{2}\theta }{\partial t^{2}}:\\ -M_{xx}+\left(M_{xx}+\Delta M_{xx}\right)-V\Delta x+N_{xx}\Delta x\frac{dw}{dx}+q(x)\Delta x(c\Delta x)=\left(\frac{1}{12}\rho bh^{3}\Delta x+\frac{1}{3}\rho hb\Delta x^{3}\right)\frac{\partial^{2}\theta }{\partial t^{2}} \end{array}$$

(10) $$N_{xx}=\int_{A^{e}}\sigma_{xx}dA\;,\;M_{xx}=\int_{A^{e}}\sigma_{xx}zdA$$

where h, b, Δx, and ρ are the thickness, width, length, and density of the beam element, respectively. Additionally, u, w, and θ are the axial displacement, transverse deflection, and slope, respectively. Furthermore, c is a constant coefficient that determine the center of the distributed transverse load in the beam element and calculate the moment of $q\left(x\right)$ about the z axis. Taking the limit Δx→0, we obtain the following equations:

(11) $$\frac{dN_{xx}}{dx}+f(x)=\rho A\frac{\partial^{2}u}{\partial t^{2}}$$

(12) $$\frac{dV}{dx}+q(x)=\rho A\frac{\partial^{2}w}{\partial t^{2}}$$

(13) $$\begin{array}{l} \frac{dM_{xx}}{dx}-V+N_{xx}\frac{dw}{dx}=\left(\frac{1}{12}\rho bh^{3}\right)\frac{\partial^{2}\theta }{\partial t^{2}}\to \\ \frac{d^{2}M_{xx}}{dx^{2}}-\frac{dV}{dx}+\frac{d}{dx}\left(N_{xx}\frac{dw}{dx}\right)=\frac{d}{dx}\left(\frac{1}{12}\rho bh^{3}\frac{\partial^{2}\theta }{\partial t^{2}}\right) \end{array}$$

Substituting Equation (12) in Equation (13), we obtain the following:

(14) $$\frac{d^{2}M_{xx}}{dx^{2}}+q(x)-\rho A\frac{\partial^{2}w}{\partial t^{2}}+\frac{d}{dx}\left(N_{xx}\frac{dw}{dx}\right)=\frac{d}{dx}\left(\frac{1}{12}\rho bh^{3}\frac{\partial^{2}\theta }{\partial t^{2}}\right)$$

Here, we derived the weak form of Equations (11) and (14) as follows:

(15) $$\begin{array}{l} 0=\int_{x_{a}}^{x_{b}}\delta u\left(-\frac{dN_{xx}}{dx}-f(x)+\rho A\frac{\partial^{2}u}{\partial t^{2}}\right)dx\\ =\int_{x_{a}}^{x_{b}}\left(\frac{d\delta u}{dx}N_{xx}-\delta uf(x)+\rho A\delta u\frac{\partial^{2}u}{\partial t^{2}}\right)dx-{\left[\delta uN_{xx}\right]}_{x_{a}}^{x_{b}}\\ =\int_{x_{a}}^{x_{b}}\left(\frac{d\delta u}{dx}N_{xx}-\delta uf(x)+\rho A\delta u\frac{\partial^{2}u}{\partial t^{2}}\right)dx+\delta u(x_{a})N_{xx}(x_{a})-\delta u(x_{b})N_{xx}(x_{b}) \end{array}$$

(16) $$\begin{array}{l} 0=\int_{x_{a}}^{x_{b}}\delta w\left[-\frac{d}{dx}\left(\frac{dw}{dx}N_{xx}\right)-\frac{d^{2}M_{xx}}{dx^{2}}-q+\rho A\frac{\partial^{2}w}{\partial t^{2}}+\frac{d}{dx}\left(\rho \frac{bh^{3}}{12}\frac{\partial^{2}\theta }{\partial t^{2}}\right)\right]dx\\ =\int_{x_{a}}^{x_{b}}\left[\frac{d\delta w}{dx}\left(\frac{dw}{dx}N_{xx}\right)-M_{xx}\frac{d^{2}\delta w}{dx^{2}}-\delta wq+\delta w\rho A\frac{\partial^{2}w}{\partial t^{2}}-\frac{d\delta w}{dx}\left(\rho \frac{bh^{3}}{12}\right)\frac{\partial^{2}\theta }{\partial t^{2}}\right]dx\\ -{\left[\delta w\frac{dw}{dx}N_{xx}+\delta w\frac{dM_{xx}}{dx}-\delta w\left(\rho \frac{bh^{3}}{12}\right)\frac{\partial^{2}\theta }{\partial t^{2}}\right]}_{x_{a}}^{x_{b}}+{\left[\frac{d\delta w}{dx}M_{xx}\right]}_{x_{a}}^{x_{b}}\\ =\int_{x_{a}}^{x_{b}}\left[\frac{d\delta w}{dx}\left(\frac{dw}{dx}N_{xx}\right)-M_{xx}\frac{d^{2}\delta w}{dx^{2}}-\delta wq+\delta w\rho A\frac{\partial^{2}w}{\partial t^{2}}-\frac{d\delta w}{dx}\left(\rho \frac{bh^{3}}{12}\right)\frac{\partial^{2}\theta }{\partial t^{2}}\right]dx\\ -\delta w(x_{b}){\left[\frac{dw}{dx}N_{xx}+\frac{dM_{xx}}{dx}-\left(\rho \frac{bh^{3}}{12}\right)\frac{\partial^{2}\theta }{\partial t^{2}}\right]}_{x_{b}}+\delta w(x_{a}){\left[\frac{dw}{dx}N_{xx}+\frac{dM_{xx}}{dx}-\left(\rho \frac{bh^{3}}{12}\right)\frac{\partial^{2}\theta }{\partial t^{2}}\right]}_{x_{a}}\\ +{\left[\frac{d\delta w}{dx}\right]}_{x_{b}}M_{xx}(x_{b})-{\left[\frac{d\delta w}{dx}\right]}_{x_{a}}M_{xx}(x_{a}) \end{array}$$

wherein, $x_{a}$ and $x_{b}$ are the beginning and end of the beam element along the x coordinate direction. Due to the nonlinear nature of the structure, we utilize a numerical solution to examine its vibration response. As a result, the axial displacement $u\left(x,t\right)$ can be found as follows:

(17) $$\begin{array}{l} u(x,t)\approx u_{1}(t)\psi_{1}(x)+u_{4}(t)\psi_{4}(x)\\ u_{1}=u\left(x,t\right),\;u_{4}=u\left(x,t\right)\\ \psi_{1}\left(x\right)=1-\frac{x-x_{a}}{L},\;\psi_{4}\left(x\right)=\frac{x-x_{a}}{L} \end{array}$$

Furthermore, the transverse deflection $w\left(x,t\right)$ can be expressed as follows:

(18) $$\begin{array}{l} w\left(x,t\right)\approx \sum_{j=2}^{3}{\bar{\Delta }}_{j}(t)\varphi_{j}\left(\bar{x}\right)+\sum_{j=5}^{6}{\bar{\Delta }}_{j}\left(t\right)\varphi_{j}\left(\bar{x}\right)\\ {\bar{\Delta }}_{2}\equiv w\left(\bar{x},t\right),\;{\bar{\Delta }}_{3}\equiv \theta \left(\bar{x},t\right),\;{\bar{\Delta }}_{5}\equiv w\left(\bar{x},t\right),\;{\bar{\Delta }}_{6}\equiv \theta \left(\bar{x},t\right)\\ \varphi_{2}\left(\bar{x}\right)=\frac{1}{L^{3}}(2{\bar{x}}^{3}-3{\bar{x}}^{2}L+L^{3}),\;\varphi_{3}\left(\bar{x}\right)=\frac{1}{L^{3}}({\bar{x}}^{3}L-2{\bar{x}}^{2}L^{2}+\bar{x}L^{3})\\ \varphi_{5}\left(\bar{x}\right)=\frac{1}{L^{3}}(-2{\bar{x}}^{3}+3{\bar{x}}^{2}L),\;\varphi_{6}\left(\bar{x}\right)=\frac{1}{L^{3}}({\bar{x}}^{3}L-{\bar{x}}^{2}L^{2}) \end{array}$$

In which, $\bar{x}=x-x_{a}$, and $\varphi_{j}$ and $\psi_{j}$ stand for the Hermite cubic and the linear Lagrange interpolation functions, respectively.

Substituting Equation (17) for $u\left(x\right)$ and $\delta u=\psi_{j}\left(x\right)$ into Equation (15), we obtain:

(19) $$\begin{array}{l} \int_{x_{a}}^{x_{b}}\left(\psi_{j}^{\prime }N_{xx}-\psi_{j}f\left(x\right)+\rho A\psi_{j}\frac{\partial^{2}u}{\partial t^{2}}\right)dx=Q_{1}\psi_{1}(x_{a})+Q_{4}\psi_{4}\left(x_{b}\right),\;for\;j=1,4\\ Q_{1}=-N_{xx}(x_{a}),\;Q_{4}=N_{xx}(x_{b}) \end{array}$$

Also, by substituting Equation (18) for $w\left(x\right)$ and $\delta w\left(x\right)=\varphi \left(x\right)$ into Equation (16), one may be write as follows:

(20) $$\begin{array}{l} \int_{x_{a}}^{x_{b}}\left(\varphi_{j}^{\prime }(\frac{dw}{dx}N_{xx})-M_{xx}\varphi_{j}^{"}-\varphi_{j}q+\varphi_{j}\rho A\frac{\partial^{2}w}{\partial t^{2}}-\varphi_{j}^{\prime }\left(\rho \frac{bh^{3}}{12}\right)\frac{\partial^{2}\theta }{\partial t^{2}}\right)dx\\ =Q_{2}\varphi_{2}(x_{a})+Q_{3}\varphi_{3}(x_{a})+Q_{5}\varphi_{5}(x_{b})+Q_{6}\varphi_{6}(x_{b})\to for\;j=2,3,5,6\\ Q_{2}=-{\left[\frac{dw}{dx}N_{xx}+\frac{dM_{xx}}{dx}-\left(\rho \frac{bh^{3}}{12}\right)\frac{\partial^{2}\theta }{\partial t^{2}}\right]}_{x_{a}}Q_{3}=-M_{xx}(x_{a})\\ Q_{5}={\left[\frac{dw}{dx}N_{xx}+\frac{dM_{xx}}{dx}-\left(\rho \frac{bh^{3}}{12}\right)\frac{\partial^{2}\theta }{\partial t^{2}}\right]}_{x_{b}}Q_{6}=M_{xx}(x_{b}) \end{array}$$

Newmark’s method is a numerical technique used to solve dynamic problems in structural analysis, particularly for vibration analysis of systems subjected to time-varying forces. It is designed to approximate the displacement and velocity of a system over time by iterating through discrete time steps. The method assumes that acceleration varies linearly between two time steps, allowing for an update of both displacement and velocity at each step based on the previous values and calculated accelerations. By using adjustable parameters, Newmark’s method can be tuned for different levels of accuracy and stability. It is widely used for solving both linear and nonlinear problems in a variety of engineering fields, including structural dynamics. This method is especially popular for its simplicity and versatility in handling complex, time-dependent behavior of structures. Accordingly, this method was chosen for our study.

Employing the Newmark method, the second-order time derivatives of axial displacement, transverse deflection, and slope can be recast as follows:

(21) $$\begin{array}{l} {\left\{\ddot{u}\right\}}_{s+1}=a_{3}\left({\left\{u\right\}}_{s+1}-{\left\{u\right\}}_{s}\right)-a_{4}{\left\{\dot{u}\right\}}_{s}-a_{5}{\left\{\ddot{u}\right\}}_{s}\\ {\left\{\ddot{w}\right\}}_{s+1}=a_{3}\left({\left\{w\right\}}_{s+1}-{\left\{w\right\}}_{s}\right)-a_{4}{\left\{\dot{w}\right\}}_{s}-a_{5}{\left\{\ddot{w}\right\}}_{s}\\ {\left\{\ddot{\theta }\right\}}_{s+1}=a_{3}\left({\left\{\theta \right\}}_{s+1}-{\left\{\theta \right\}}_{s}\right)-a_{4}{\left\{\dot{\theta }\right\}}_{s}-a_{5}{\left\{\ddot{\theta }\right\}}_{s}\\ a_{3}=\frac{1}{\beta {\left(\Delta t\right)}^{2}},\;a_{4}=a_{3}\Delta t,\;a_{5}=\frac{1}{\gamma }-1 \end{array}$$

(22) $$\begin{array}{l} {\left\{\dot{u}\right\}}_{s+1}={\left\{\dot{u}\right\}}_{s}+a_{2}{\left\{\ddot{u}\right\}}_{s}+a_{1}{\left\{\ddot{u}\right\}}_{s+1}\\ {\left\{\dot{w}\right\}}_{s+1}={\left\{\dot{w}\right\}}_{s}+a_{2}{\left\{\ddot{w}\right\}}_{s}+a_{1}{\left\{\ddot{w}\right\}}_{s+1}\\ {\left\{\dot{\theta }\right\}}_{s+1}={\left\{\dot{\theta }\right\}}_{s}+a_{2}{\left\{\ddot{\theta }\right\}}_{s}+a_{1}{\left\{\ddot{\theta }\right\}}_{s+1}\\ a_{1}=\alpha \Delta t,\;a_{2}=\left(1-\alpha \right)\Delta t \end{array}$$

where $\left\{\ddot{u}\right\}$, $\left\{\ddot{w}\right\}$, and $\left\{\ddot{\theta }\right\}$ are the second-order time derivatives of axial displacement, transverse deflection, and slope, respectively. Furthermore, $\left\{\dot{u}\right\}$, $\left\{\dot{w}\right\}$, and $\left\{\dot{\theta }\right\}$ are the first-time derivatives of axial displacement, transverse deflection, and slope, respectively. Moreover, Δt is the amount of time increments and S shows the number of time steps. Additionally, α, β, and γ are parameters that determine the stability of the time integration scheme. In this work, the constant average acceleration method was implemented; thus, the amount of these parameters is as follows:

(23) $$\alpha =\frac{1}{2},\;\gamma =2\beta =\frac{1}{2}$$

To solve this problem, we divided the height and length of the beam into uniform parts. At each cross-section, we have to solve Equations (19) and (20) to calculate the axial displacement, transverse deflection, and slope. These parameters are coupled in every cross-section. Hence, in order to solve Equations (19) and (20), we assembled them into a universal matrix in each iteration.

**Figure 2:**
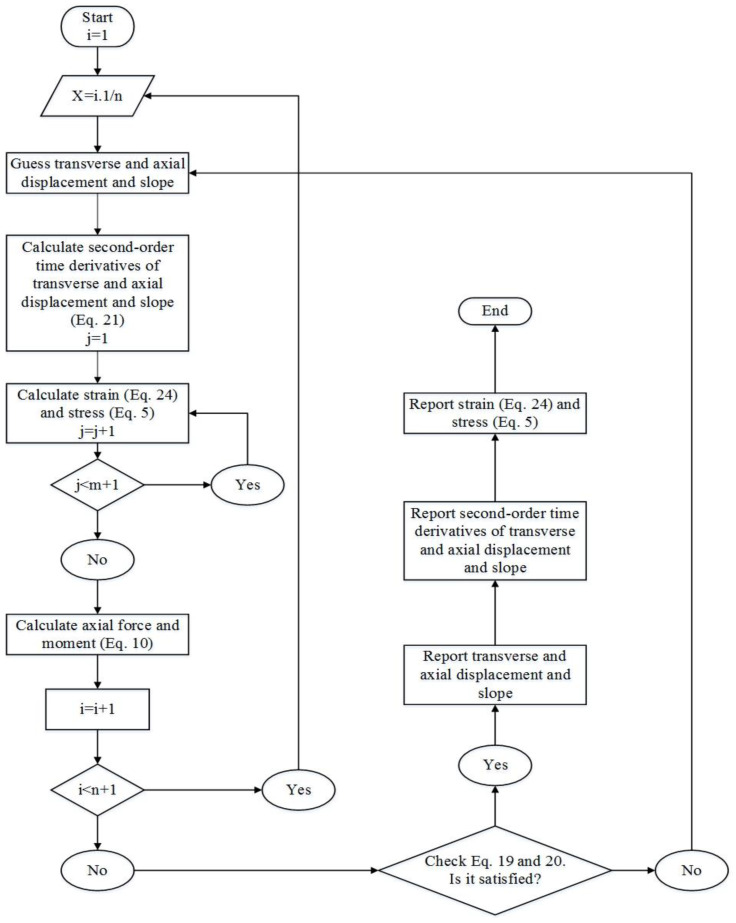
Solution algorithm for the proposed formulation.

As depicted in Figure 2, a flowchart is implemented to interpret the solution procedure. First, we choose an apt initial guess (reference configuration) for $u\left(x\right)$, $w\left(x\right)$, and $\theta \left(x\right)$ at each cross-section, and by substituting these parameters in Equation (24), one may achieve the amount of strain.

(24) $$\begin{array}{l} \varepsilon_{11}=\varepsilon_{xx}=\frac{du_{0}}{dx}-z\frac{d^{2}w_{0}}{dx^{2}}=\left(\frac{du_{0}}{dx}\right)-z\left(\frac{d^{2}w_{0}}{dx^{2}}\right)=\varepsilon_{xx}^{0}+z\varepsilon_{xx}^{1}\\ \varepsilon_{xx}^{0}=\frac{du_{0}}{dx}\;,\;\varepsilon_{xx}^{1}=-\frac{d^{2}w_{0}}{dx^{2}} \end{array}$$

These strain definitions are known as the Von Kármán strains. They are a set of nonlinear strain–displacement relations used in structural mechanics to describe moderately large deformations in thin plates and beams. They extend classical linear strain theory by incorporating nonlinear terms that account for geometric nonlinearities, making them particularly useful for problems involving moderate rotations while still assuming small strains. These relations are employed in the finite element formulations of this work.

Subsequently, when the input variables in PM are the strain and temperature, we can compute the stress in each node by implementing a numerical method. Then, we calculate the axial force and moment by Equation (10). Finally, the Newton–Raphson method is implemented to solve Equations (19) and (20). Therefore, if the initial values for $u\left(x\right)$, $w\left(x\right)$, and $\theta \left(x\right)$ do not satisfy Equations (19) and (20), the Newton–Raphson algorithm will continue until meeting an acceptable tolerance. It is noteworthy that, in this model, we implement the Newton–Raphson numerical method in both the beam model and PM. By employing this numerical procedure, accurate answers can be found in a short time.

Due to the complication of the proposed FEM procedure, a concise explanation is provided here. First, a beam with any layup should be divided into a reasonable number of elements. Then, the load vector and boundary conditions should be imposed to the FEM equation ($\left\{F\right\}=\left[K\right]\left\{U\right\}$), which is formed by assembling Equations (19) and (20) for each element. Furthermore, $\left[K\right]$ and $\left\{U\right\}$ are the matrix of global stiffness and vector of global displacement, respectively. For materials like SMAs whose stiffness is history-dependent, the matrix of stiffness $\left[K\right]$ is not constant and is dependent on the applied force. Due to the fact that $\left[K\right]$ depends on both $\left\{U\right\}$ and $\left\{F\right\}$, it is impossible to calculate $\left\{U\right\}$ without using an iterative procedure. To this end, firstly, we need to choose an apt initial guess for $\left\{U\right\}$, and then calculate $\left[K\right]$ which is dependent on $\left\{U\right\}$. We continue this procedure (by using Newton–Raphson relations) until we find an accurate value of $\left\{U\right\}$ for any specified $\left\{F\right\}$. After the solution converged in the first iteration, the results of the previous step are utilized as the new initial guess for the next iteration. Thus, a new guess is not required for each iteration.

It needs to be mentioned that the Newton–Raphson method is a powerful numerical technique used to find the roots or solutions of nonlinear equations. It is an iterative process that starts with an initial guess and refines it by approximating the function with a tangent line at each step. At each iteration, the method calculates the next approximation by using the value of the function and its derivative at the current point. This process continues until the solution converges to a sufficiently accurate value. The method is widely used in solving systems of nonlinear equations. Its primary advantage is its rapid convergence, especially when the initial guess is close to the true solution.

## 4. Numerical Results

In this section, several numerical examples are represented to evaluate the performance of the presented model. SMA materials that are used in this work are entitled as $M_{1}$ and $M_{2}$. These materials are examined by Gall et al. [51] and Flor et al. [50], respectively. The material parameters listed in Table 1 and Table 2 are elicited by Poorasadion et al. [17]. In all examples, the intensity of the external force is chosen high enough to bring about the phase transformation to observe SMA’s unique behavior. On the other hand, this force should not be too high to induce large deflections and accordingly contradict the Euler–Bernoulli beam theory assumptions.

### 4.1. Validation

In this section, the results of the proposed model are verified with the aid of numerical results achieved by the finite element software ABAQUS/Standard for a homogenous SMA beam. To capture the vibration behavior of this specific problem, a user-defined material subroutine UMAT in ABAQUS [17] is utilized. In addition, by considering a 2D Euler–Bernoulli beam element, the vibration response of a homogeneous SMA beam is simulated. It is noteworthy to mention that by employing a UMAT subroutine in ABAQUS software, we can only model a homogeneous SMA beam.

**Figure 3:**
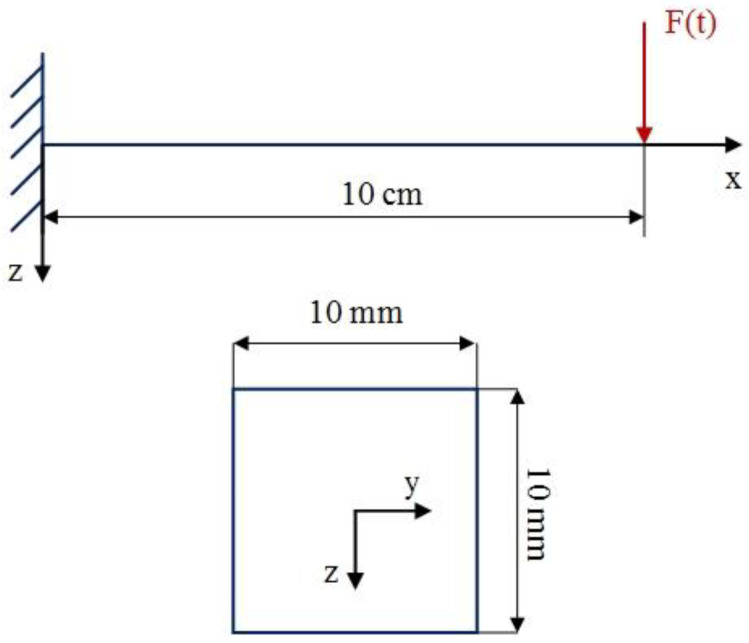
Geometry of the beam and the cross-section.

**Figure 4:**
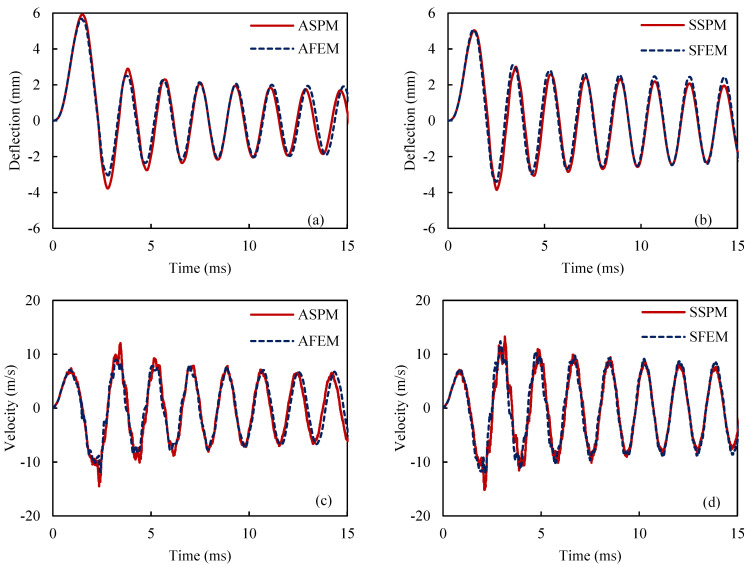
History of the maximum deflection (**a**,**b**) and velocity (**c**,**d**) for the ASYM and SYM models and also for the proposed model and finite element simulations.

For this purpose, an SMA beam with the geometry indicated in Figure 3, is considered. The beam is fixed in one end $\left(z\left(x=0\right)=0\;and\;{\left.\frac{dz}{dx}\right|}_{x=0}=0\right)$ and it is free on the other end. These boundary conditions are applied to all beams described in this work. However, the applied forces vary and are specified individually for each example. The selected boundary conditions are equal to a fixed-free beam which is a good representative for structural applications. Additionally, at the beginning of the simulation, all beam elements are initialized with zero stresses, strains, displacement, and velocity. This approach remains consistent throughout all examples in this work. To analyze the vibration response of the SMA beam, a dynamic load is applied at the free tip of the beam. A mesh convergence analysis is conducted to find the suitable size and number of elements. Thus, the SMA beam includes 30 elements in both methods. Figure 4 represents the time history of the maximum deflection and velocity of the SMA beam, made of $M_{2}$ material, where the density of the material is 6500 $kg/m^{3}$, and the temperature is +83 °C$\left(>A_{f}\right)$ in which the behavior of the material is superelastic. Dynamic forces are applied vertically to the free end of the described beam. The intensity of the force is expressed as follows:

(25) $$F=\left(\begin{array}{lll} \left(625\frac{t}{0.0009}\right)\;N & \; & \mathrm{if}\;0\leq t\leq 0.0009\;(s)\\ \left(625\left(2-\frac{t}{0.0009}\right)\right)\;N & \; & \mathrm{if}\;0.0009\leq t\leq 0.0018\;(s) \end{array}\right.$$

In Figure 4, ASPM, AFEM, SSPM, and SFEM stand for the ASYM proposed model, the ASYM finite element model, the SYM proposed model, and the SYM finite element model, respectively. Throughout the article, model names beginning with “A” indicate an asymmetric material behavior assumption, while those starting with “S” represent a symmetric material behavior assumption. As shown Figure 4, the results of the proposed model are in good agreement with the numerical results from ABAQUS/Standard (with a 3% error). Nevertheless, there is a noticeable difference between the forecasting ASPM and SSPM models. Regarding Figure 4a,b, the energy dissipation effect as well as the vibration amplitude reduction is obvious, which stems from the hysteresis property of SMA materials.

### 4.2. Free Vibration Behavior of a Tri-Layer SMA Beam

**Figure 5:**
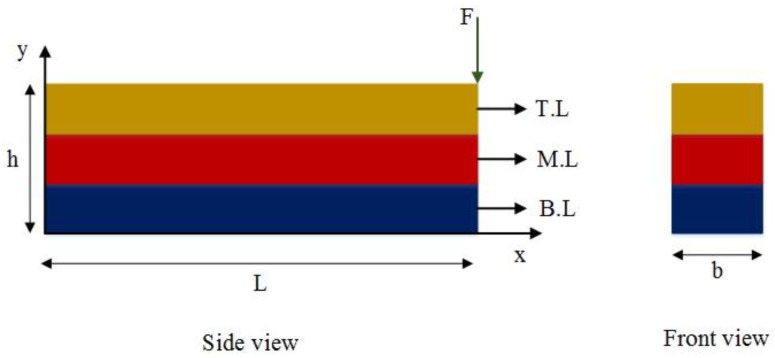
Schematic of the tri-layer composite beam.

In this section, two numerical examples are presented to analyze the free vibration behavior of a tri-layer SMA composite beam. Regarding Figure 5, the top, middle, and bottom layers of the beam are made of $M_{1}$, $M_{2}$ and $M_{1}$, respectively. The geometry of the clamped composite beam is the same as the previous model. For both examples, the thickness of the top and bottom layers of the beam is 2.5 mm, and the thickness of the middle layer is 5 mm. The temperature is +83 °C$\left(>A_{f}\right)$ to trigger the superelastic behavior in both materials.

#### 4.2.1. Free Vibration of the Tri-Layer SMA Composite Beam

In order to analyze the free vibration behavior of the tri-layer SMA beam, a dynamic load is applied at the free tip of the beam. The intensity of the force is introduced as follows:

(26) $$F=\left(\begin{array}{lll} \left(750\frac{t}{0.0009}\right)\;N & \; & \mathrm{if}\;0\leq t\leq 0.0009\;(s)\\ \left(750\left(2-\frac{t}{0.0009}\right)\right)\;N & \; & \mathrm{if}\;0.0009\leq t\leq 0.0018\;(s) \end{array}\right.$$

**Figure 6:**
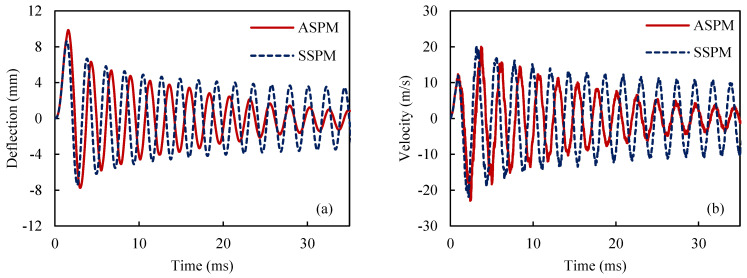

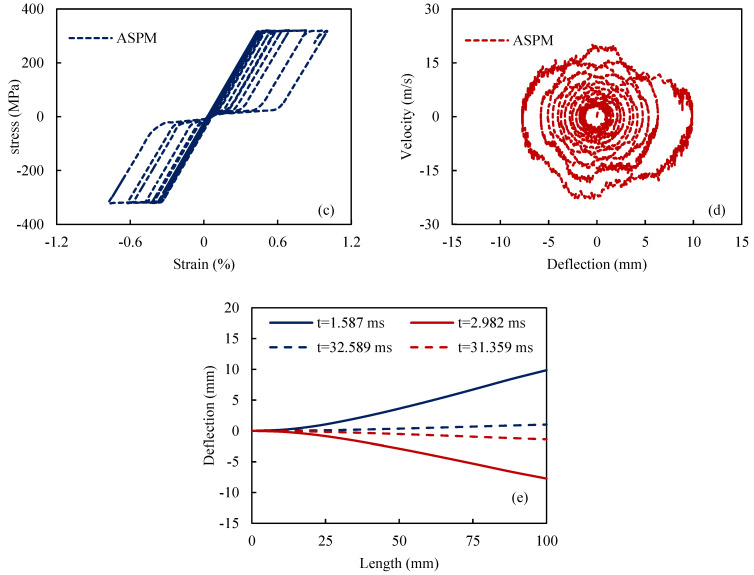
Free vibration analysis of tri-layer SMA composite beam, (**a**) time history of the tri-layer beam tip deflections, (**b**) time history of the velocity of the free end of the beam, (**c**) strain–stress diagram of a point with 5 mm distance from the clamped end of the beam, (**d**) phase portrait of the free end of the beam, (**e**) deflection of the beam four individual times.

Figure 6a plots the tip deflection of the tri-layer SMA composite beam. It is evident from Figure 6a that the vibration amplitude of the beam reduced effectively a short time after the beginning of the oscillation. The reason behind this reduction is SMA’s hysteresis property. The steady-state part of the results indicates that the SMA composite beam vibrates as an elastic material. Figure 6b illustrates the velocity-time diagram of the free tip of the composite beam. Regarding Figure 6a,b, there is a noticeable deviation between the ASPM and SSPM models, which indicates the importance of considering the asymmetric behavior of these materials. In order to understand the state of phase transformation inside the SMA composite beam, Figure 6c plots the stress–strain diagram of a point with a 5 mm distance from the clamped root of the beam. It is apparent from Figure 6c that at the start of the oscillation, the hysteresis loop is formed, and then steadily, this loop shrinks. Finally, the hysteresis loop takes a linear form. As a result, the SMA beam loses its energy-dissipating behavior. The phase diagram in Figure 6d shows the behavior of the free tip of the composite beam for both transient and steady-state. In the transient state, some chaotic behavior is observed; though, in the steady state, this chaotic behavior fades away because of the SMA’s hysteresis behavior. Figure 6e depicts the deflection of the free end of the tri-layer SMA beam at four different moments, where two of them are at the transient time, and the other two are at the steady-state.

**Figure 7:**
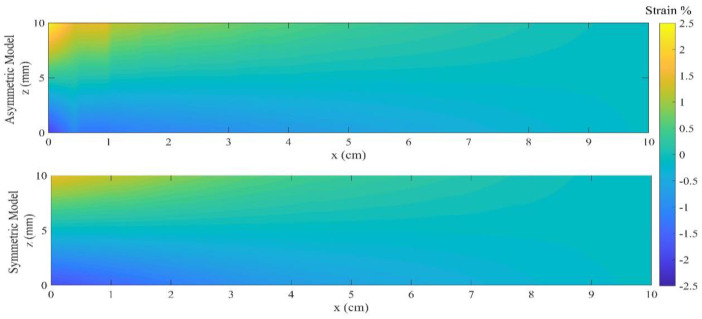
Contour plot of strain for ASPM and SSPM models.

The contour plot for the strain at the maximum loading is illustrated in Figure 7, where the maximum strain in tension for ASPM and SSPM models are $\varepsilon_{\max}^{t}=2.45\;(\%)$ and $\varepsilon_{\max}^{t}=1.9\;(\%)$, respectively. Moreover, the maximum strain in compression for ASPM and SSPM models are $\varepsilon_{\max}^{c}=2.1\;(\%)$ and $\varepsilon_{\max}^{c}=1.7\;(\%)$, respectively.

#### 4.2.2. Parametric Study

**Figure 8:**
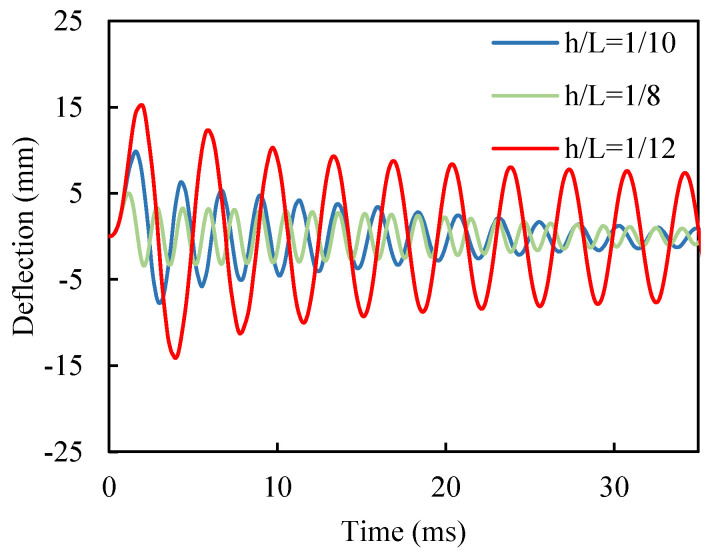
Size parameter effect on time history of the tri-layer beam tip deflections for size parameter $\frac{h}{L}=\frac{1}{8}$, $\frac{h}{L}=\frac{1}{10}$, and $\frac{h}{L}=\frac{1}{12}$.

A size parameter defined as the ratio of beam thickness to its length ($\frac{h}{L}$), is utilized in this study to analyze the influence of beam length on the system vibration. Three different amplitude of size parameters ($\frac{h}{L}=\frac{1}{8}$, $\frac{h}{L}=\frac{1}{10}$, and $\frac{h}{L}=\frac{1}{12}$) have been assumed to examine the dynamic behavior of the tri-layer SMA beam. For all three cases, the initial conditions and all required parameters are the same as the previous model. Figure 8 plots the free vibration of the tri-layer SMA beam for three different values of size parameters. As Figure 8 indicates, the steady-state amplitude amplifies with longer beams. For example, the steady-state amplitude is 1.1, 1.9, and 6.5 mm for $\frac{h}{L}=\frac{1}{8}$, $\frac{h}{L}=\frac{1}{10}$, and $\frac{h}{L}=\frac{1}{12}$**,** respectively. In other words, the thinner beam performs less chaotic in comparison to the thicker ones. The results indicate that, for a thinner beam, phase transformation of constituent elements of the beam (the primary mechanism behind damping) occurs less frequently compared to thicker beams. As a result, when the vibration reaches a steady state, the vibration amplitude in thinner beams remains relatively higher. In practical structural applications, it is crucial to consider that the composite beam’s layup and layer arrangement should be optimized based on the applied forces. This ensures that phase transformation occurs efficiently; otherwise, the beam may behave like a conventional beam without significant damping properties.

### 4.3. Free Vibration Behavior of a Bi-Layer SMA Beam

**Figure 9:**
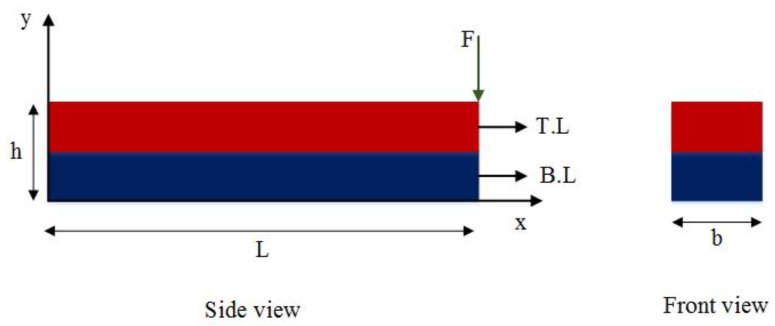
Schematic of the bi-layer SMA beam.

In this section, two numerical examples are presented to analyze the free vibration response of bi-layer SMA composite beam. Regarding Figure 9, the top and bottom layers of the bilayer composite beam are respectively made of $M_{1}$ and $M_{2}$. The geometry of the clamped composite beam is the same as the previous model. For both examples, the thickness of each layer of the composite beam is 5 mm. The temperature is +83 °C$\left(>A_{f}\right)$ to activate the superelastic behavior of both materials.

#### 4.3.1. Free Vibration of the Bi-Layer SMA Composite Beam

In order to analyze the free vibration response of the bi-layer SMA beam, a dynamic load is applied at the free end of the beam. The intensity of the force is selected as follows:

(27) $$F=\left(\begin{array}{lll} \left(750\frac{t}{0.0009}\right)\;N & \; & \mathrm{if}\;0\leq t\leq 0.0009\;(s)\\ \left(750\left(2-\frac{t}{0.0009}\right)\right)\;N & \; & \mathrm{if}\;0.0009\leq t\leq 0.0018\;(s) \end{array}\right.$$

**Figure 10:**
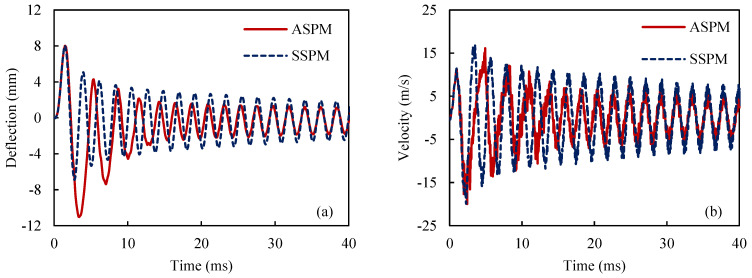

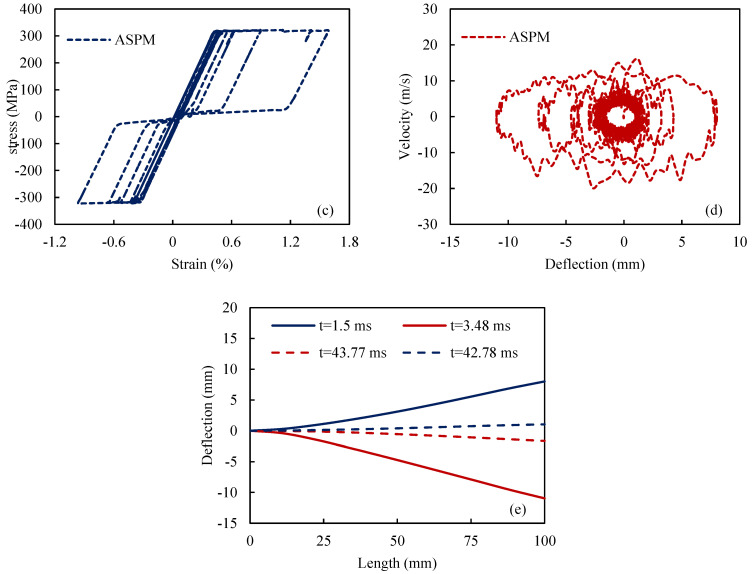
Free vibration analysis of bi-layer SMA composite beam, (**a**) time history of the bi-layer beam tip deflections, (**b**) time history of the velocity of the free end of the beam, (**c**) strain–stress diagram of a point with 5 mm distance from the clamped end of the beam, (**d**) phase portrait of the free end of the beam, (**e**) deflection of the beam four individual times.

Figure 10a plots the free tip deflection of the bi-layer SMA composite beam. From Figure 10a it is apparent that the vibration amplitude is reduced effectively a short time after the beginning of the vibration stemming from SMA’s hysteresis property. The steady-state part of the results reveals that the SMA composite beam vibrates as an elastic material. Figure 10b illustrates the velocity–time diagram of the free end of the composite beam. Comparing the data shown in Figure 10a,b, a noticeable difference is observed between the ASPM and SSPM models, which justifies the importance of considering the asymmetric behavior of these materials. To better understand the state of phase transformation inside the SMA composite beam, Figure 10c plots the stress–strain diagram of a point with a 5 mm distance from the beam root. The slope of the stress–strain diagram of the clamped end is very close to zero when the material enters to the phase transformation regime; thus, the material becomes softer, and as a consequence, larger amplitudes, and strains are achieved. The phase diagram in Figure 10d shows the behavior of the beam tip for both transient and steady-state solutions. In the transient state, some chaotic behavior is revealed; though, in the steady-state, due to the SMA’s hysteresis response, this chaotic behavior disappears. Figure 10e also illustrates the deflection of the free end of the tri-layer SMA beam at four different moments where the first two of them are at the transient response regime, and the last two are at the steady-state.

**Figure 11:**
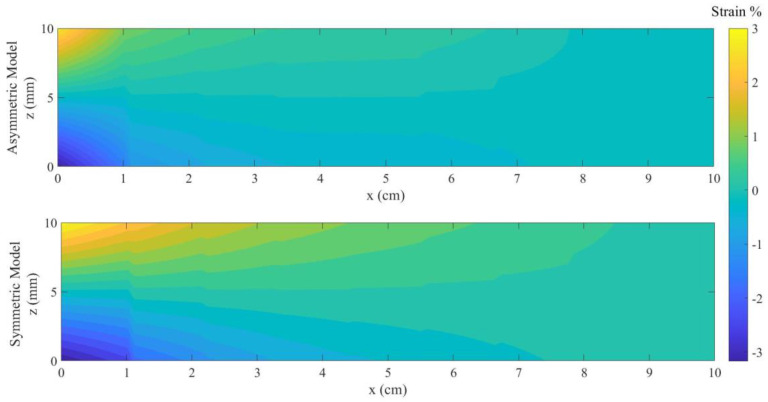
Contour plots of the strain for ASPM and SSPM models.

The contour plot for the strain at the maximum loading point is illustrated in Figure 11, where the maximum strain in tension for ASPM and SSPM models are $\varepsilon_{\max}^{t}=3.1\;(\%)$ and $\varepsilon_{\max}^{t}=2.3\;(\%)$, respectively. In addition, the maximum strain in compression for ASPM and SSPM models are $\varepsilon_{\max}^{c}=2.9\;(\%)$ and $\varepsilon_{\max}^{c}=2.1\;(\%)$, respectively.

#### 4.3.2. Effect of Loading Amplitude on Free Vibration Response

In this section, the influence of the amplitude of the impulse loading on the dynamic behavior of the beam is investigated. For this purpose, the free vibration of the bilayer SMA beam under two different intensities of loading is analyzed. The intensity of the force is assumed as follows:

(28) $$F=\left(\begin{array}{lll} \left(B\frac{t}{0.0009}\right)\;N & \; & \mathrm{if}\;0\leq t\leq 0.0009\;(s)\\ \left(B\left(2-\frac{t}{0.0009}\right)\right)\;N & \; & \mathrm{if}\;0.0009\leq t\leq 0.0018\;(s) \end{array}\right.\;B=750\;N\text{ \& }B=600\;N$$

**Figure 12:**
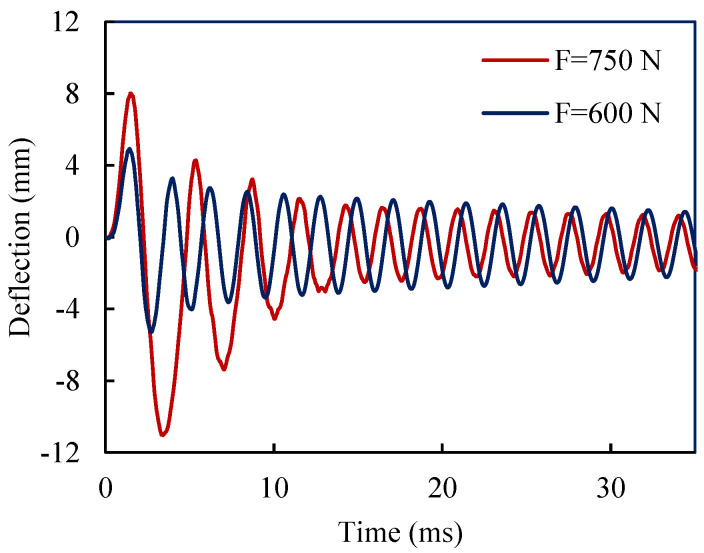
Time history of the bi-layer beam tip deflections for different amplitude of impulse loading.

Figure 12 depicts the tip deflection of the bi-layer SMA composite beam for two impulsive loads. As shown in Figure 12, due to the vibration response of the bilayer SMA beam, the rate of decay of vibration under a larger loading amplitude is higher. In other words, the SMA layers show higher capability in dissipating energy and damping the vibration of the bilayer beam under a larger intensity of loading. The underlying reason for this behavior is that SMA elements under larger force can dissipate more energy; obviously, up to a certain point when the material has completed its transformation.

## 5. Concluding Remarks

In this article, a finite element solution for examining the vibration of a multi-layer SMA composite beam was developed. The proposed model in this work offers a flexible tool for more accurate prediction of the vibration behavior of such beams. The main difference between this model with the ones available in the literature is that this model can capture the asymmetric material behavior which the results show has a considerable effect on the results of the vibration behavior. Moreover, the complicated multilayer beam problem is formulated with a simple 2D beam element in which by only utilizing the integration points (assigning different material if necessary for each integration point) the composite beam is simulated. It is further mentioned that the interaction between different layers is not considered here. Additionally, the proposed model is only capable of predicting the material behavior of an Euler–Bernoulli beam and further investigations needs to be performed for more complicated beam assumptions like Timoshenko beam. The introduced structural component in this work can be utilized in various industries. For example, it can help minimize vibrations in aircraft components, improving stability and performance or it may be employed in robotics to reduce unwanted oscillations, ensuring smoother operation and extended lifespan of mechanical systems.

By observing the response of multi-layer SMA beam for different conditions, the following results are achieved:

1. Due to the hysteresis property of SMAs, the vibration amplitude of the beam is reduced effectively a short time after the beginning of the oscillation.
2. In the steady-state part of the solution, the SMA composite beam vibrates as an elastic material.
3. Regarding the tip deflection and the velocity–time diagrams, there is a noticeable deviation between the asymmetric and symmetric models.
4. Due to the energy-dissipating behavior of SMAs, the hysteresis loop in the stress–strain diagram shrinks steadily until it takes a linear form.
5. Regarding the phase diagram, the transient state and steady state are observed.
6. Performing a parametric study on the multi-layer SMA beam, the results revealed that the steady-state amplitude rises in a smaller height to length ratio.
7. SMA layers show higher capability in damping the vibration and dissipating energy of the multi-layer beam under a larger intensity of loading.

## Figures and Tables

**Table 1:** Parameters of PM for Gall et al. [51] experiment.

	ASYM Model	SYM Model
Modulus [GPa]	$D_{m}^{+}=11,\;D_{m}^{-}=80,\;D_{m}^{T}=11,{\;D}_{a}=39$	$D_{m}^{\pm }=11,\;D_{m}^{T}=11,{\;D}_{a}=39$
Stresses [MPa]	$\sigma_{s}^{+}=60,\;\sigma_{f}^{+}=160,\;\sigma_{s}^{-}=5,\;\sigma_{f}^{-}=260$	$\sigma_{s}^{\pm }=60,\;\sigma_{f}^{\pm }=160$
Slopes [MPa/°C]	$C_{M}^{+}=8,\;C_{A}^{+}=3.5,\;C_{M}^{-}=5,\;C_{A}^{-}=3.5$	$C_{M}^{\pm }=8,\;C_{A}^{\pm }=3.5$
Strains [-]	$\varepsilon_{L}^{+}=0.034,\;\varepsilon_{L}^{-}=-0.021$	$\varepsilon_{L}^{\pm }=\pm 0.034$
Temperatures [°C]	$A_{f}=45,A_{s}=30,\;M_{s}=-3,\;M_{f}=-10$	

**Table 2:** Parameters of PM for Flor et al. [50] experiment.

	ASYM Model	SYM Model
Modulus [GPa]	$D_{m}^{+}=30,\;D_{m}^{-}=40,\;D_{m}^{T}=35,{\;D}_{a}=76.8$	$D_{m}^{\pm }=30,\;D_{m}^{T}=35,{\;D}_{a}=76.8$
Stresses [MPa]	$\sigma_{s}^{+}=50,\;\sigma_{f}^{+}=90,\;\sigma_{s}^{-}=103,\;\sigma_{f}^{-}=104$	$\sigma_{s}^{\pm }=50,\;\sigma_{f}^{\pm }=90$
Slopes [MPa/°C]	$C_{M}^{+}=7.8,\;C_{A}^{+}=4,\;C_{M}^{-}=3.38,\;C_{A}^{-}=4$	$C_{M}^{\pm }=7.8,\;C_{A}^{\pm }=4$
Strains [-]	$\varepsilon_{L}^{+}=0.074,\;\varepsilon_{L}^{-}=-0.045$	$\varepsilon_{L}^{\pm }=\pm 0.074$
Temperatures [°C]	$A_{f}=82.5,\;A_{s}=75,M_{s}=49,\;M_{f}=24$	

## Data Availability

The original contributions presented in this study are included in the article. Further inquiries can be directed to the corresponding author.
